# Spatiotemporal Control Over Circadian Rhythms With Light

**DOI:** 10.1002/med.22099

**Published:** 2025-01-05

**Authors:** Dušan Kolarski, Wiktor Szymanski, Ben L. Feringa

**Affiliations:** ^1^ Max Planck Institute for Multidisciplinary Sciences NanoBioPhotonics Göttingen Germany; ^2^ Centre for Systems Chemistry, Stratingh Institute for Chemistry University of Groningen Groningen The Netherlands; ^3^ Department of Radiology, Medical Imaging Center University Medical Center Groningen, University of Groningen Groningen The Netherlands; ^4^ Department of Medicinal Chemistry, Photopharmacology and Imaging, Groningen Research Institute of Pharmacy University of Groningen Groningen The Netherlands

**Keywords:** azobenzene, circadian clock, circadian rhythm, light, photopharmacology, photo‐removable protecting group, photoswitch

## Abstract

Circadian rhythms are endogenous biological oscillators that synchronize internal physiological processes and behaviors with external environmental changes, sustaining homeostasis and health. Disruption of circadian rhythms leads to numerous diseases, including cardiovascular and metabolic diseases, cancer, diabetes, and neurological disorders. Despite the potential to restore healthy rhythms in the organism, pharmacological chronotherapy lacks spatial and temporal resolution. Addressing this challenge, chrono‐photopharmacology, the approach that employs small molecules with light‐controlled activity, enables the modulation of circadian rhythms when and where needed. Two approaches—relying on irreversible and reversible drug activation—have been proposed for this purpose. These methodologies are based on photoremovable protecting groups and photoswitches, respectively. Designing photoresponsive bioactive molecules requires meticulous structural optimization to obtain the desired chemical and photophysical properties, and the design principles, detailed guidelines and challenges are summarized here. In this review, we also analyze all the known circadian modulators responsive to light and dissect the rationale following their construction and application to control circadian biology from the protein level to living organisms. Finally, we present the strength of a reversible approach in allowing the modulation of the circadian period and the phase.

## 1 Introduction

### 1.1 Origin and Hierarchy of Circadian Rhythms

**Figure 1:**
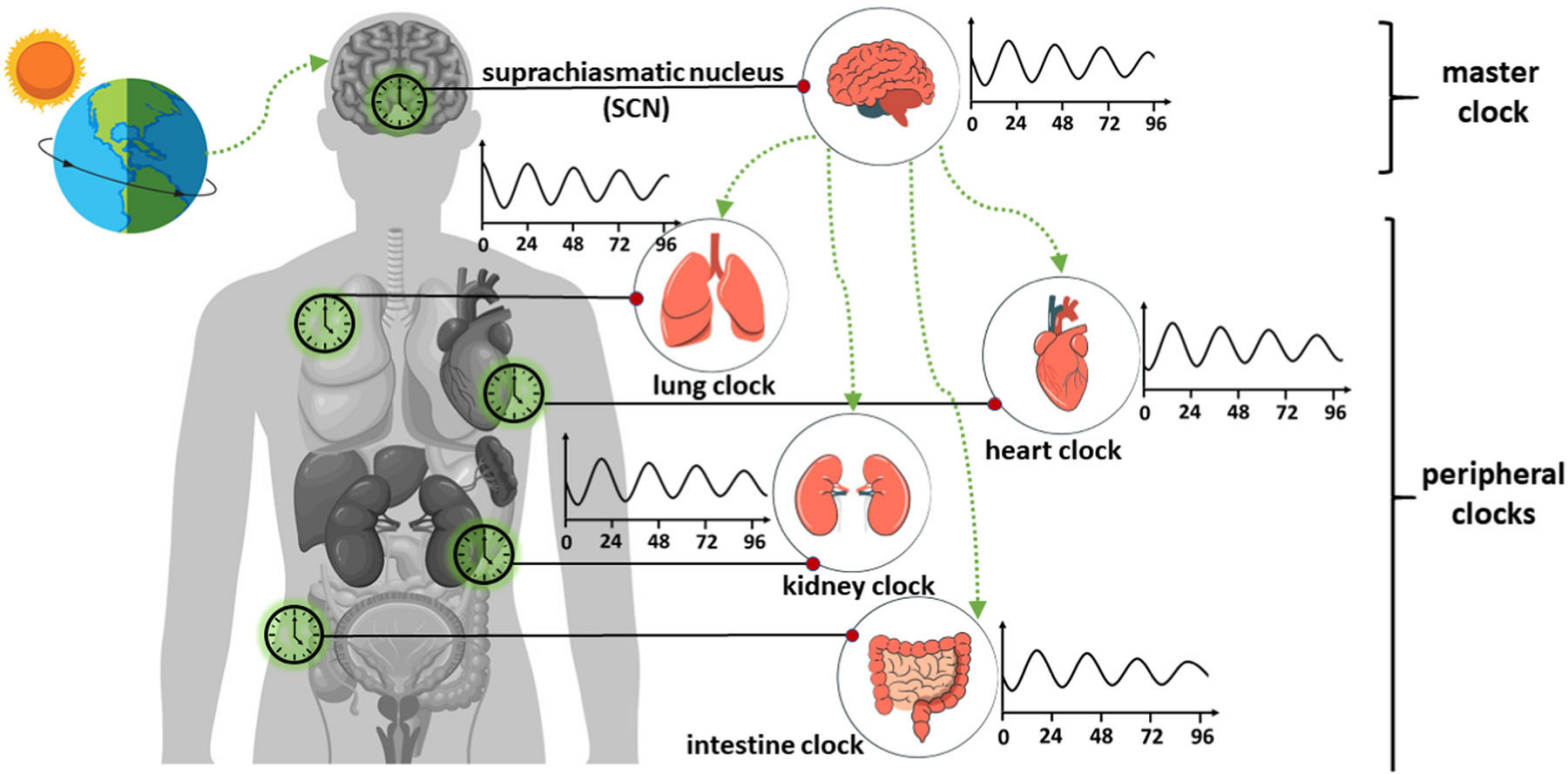
Origin and hierarchical organization of circadian rhythms. Parts of the figure were downloaded from www.freepik.com and modified accordingly.

To survive and retain healthy homeostasis, living organisms must adapt to significant daily environmental changes (e.g., light and temperature) caused by Earth's rotation around its axis. Consequently, nearly all living organisms, from bacteria to humans, possess an intrinsic time‐keeping system (circadian clock) that aligns daily biological rhythms (circadian rhythms) with Earth's cycles [1]. These cellular clocks govern the synchronization of internal physiological processes and behavior with external rhythmical changes (Figure 1) [2]. At a biochemical level, circadian clocks are robust endogenous biological oscillators with a period of approximately (“circa,” in Latin) 1 day (“dies”).

The clocks are hierarchically organized into a “master” clock and peripheral clocks (Figure 1). Light acts as a primary *zeitgeber* (“time giver” or “synchronizer“) by being detected in the eyes through intrinsically photoreceptive retinal ganglion cells (ipRGCs) that respond to blue light [3]. The signal is further transmitted to the “master” clock or the circadian pacemaker. The master clock is located in the suprachiasmatic nucleus (SCN), a 20,000‐neurons‐large bilateral region situated in the hypothalamus, just over the optic chiasm—the part of the brain where the nerves of the eyes cross [4]. Carrying information about astronomical time, SCN synchronizes the rhythms of peripheral clocks throughout the body [5]. Peripheral clocks are endogenous cellular rhythms in organs and tissues different from SCN. They have a uniform circadian period of approximately 24 h and varying phases that adjust the timing of localized processes according to the time of day [6].

### 1.2 Circadian Rhythms in Health, Disorder, and Diseases

In mammals, clock genes and protein expression rhythms occur in almost all cells [7, 8]. According to Hogenesch et al., 43% of all protein‐coding genes show circadian oscillation in at least one mouse tissue, while Mure et al. demonstrated that this number is > 80% in primates [9, 10]. Notably, > 82% of genes coding for druggable proteins (identified by the US Food and Drug Administration) have daily rhythms, indicating the importance of understanding and controlling these oscillations [9, 10].

**Figure 2:**
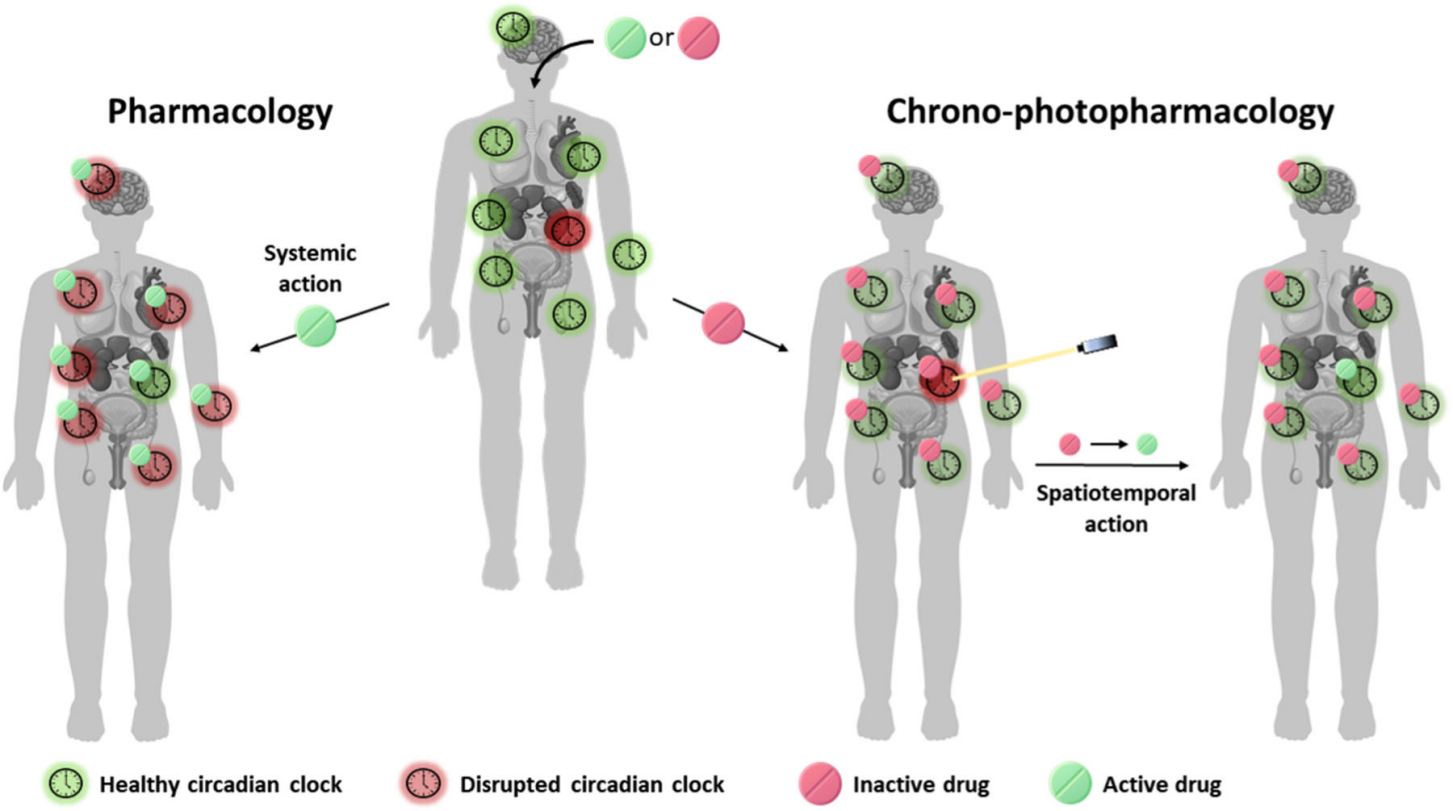
Disruption of a peripheral circadian clock and two approaches to restoring healthy rhythms: pharmacological (systemic action) and chrono‐photopharmacological (spatiotemporal action). Parts of the figure were downloaded from www.freepik.com and modified accordingly.

The master clock primarily regulates general functions such as mood and behavior, while peripheral clocks manage specific functions like hormone secretion, metabolism, heart rate, and body temperature [11, 12, 13]. Constant desynchrony from environmental cues (due to light pollution, night shifts, obesity, transmeridian travel, etc.) or genetic mutations in clock genes disrupt circadian homeostasis, leading to organ‐specific diseases or physiological disruption (Figure 2) [2, 8, 14, 15, 16, 17, 18, 19]. Desynchronization of biological time with external day‐night cycles primarily disrupts peripheral circadian clocks, and it is associated with numerous diseases, including cardiovascular and metabolic diseases, cancer, diabetes, neurological disorders (autism, depression, Parkinson's), jet lag, and so forth [2, 14, 20, 21, 22, 23, 24, 25]. Ablating the pancreatic clock in mice results in severe glucose intolerance and diabetes mellitus [26, 27]. In Alzheimer's disease, circadian rhythm phases are dysregulated across various brain regions [28], while Rijo‐Ferreira et al. discovered that sleeping sickness, caused by *Trypanosoma brucei*, is a circadian disorder [29]. The dampening amplitude of the circadian oscillations has also been linked to aging [30]. The prevalence of diseases related to circadian rhythm disruption underlines the critical need for chronotherapy [31].

### 1.3 Chronotherapy: Drugging the Circadian Clock

Optimizing the circadian lifestyle (“training the clock”) and timing of drug administration (“clocking the drugs”) can positively influence the restoration of healthy circadian rhythms and increase the effectiveness of drugs, respectively [32]. Nevertheless, when metabolic or signaling pathways are disrupted at the protein or gene level, time‐restricted feeding or following natural rhythms cannot restore proper functioning. Thus, pharmacologically interfering with the dysregulated circadian clock administration (‘drugging the clock’) through regulatory components may restore homeostasis, thereby preventing the development of chronic diseases and disorders (Figure 2) [33].

**Figure 3:**
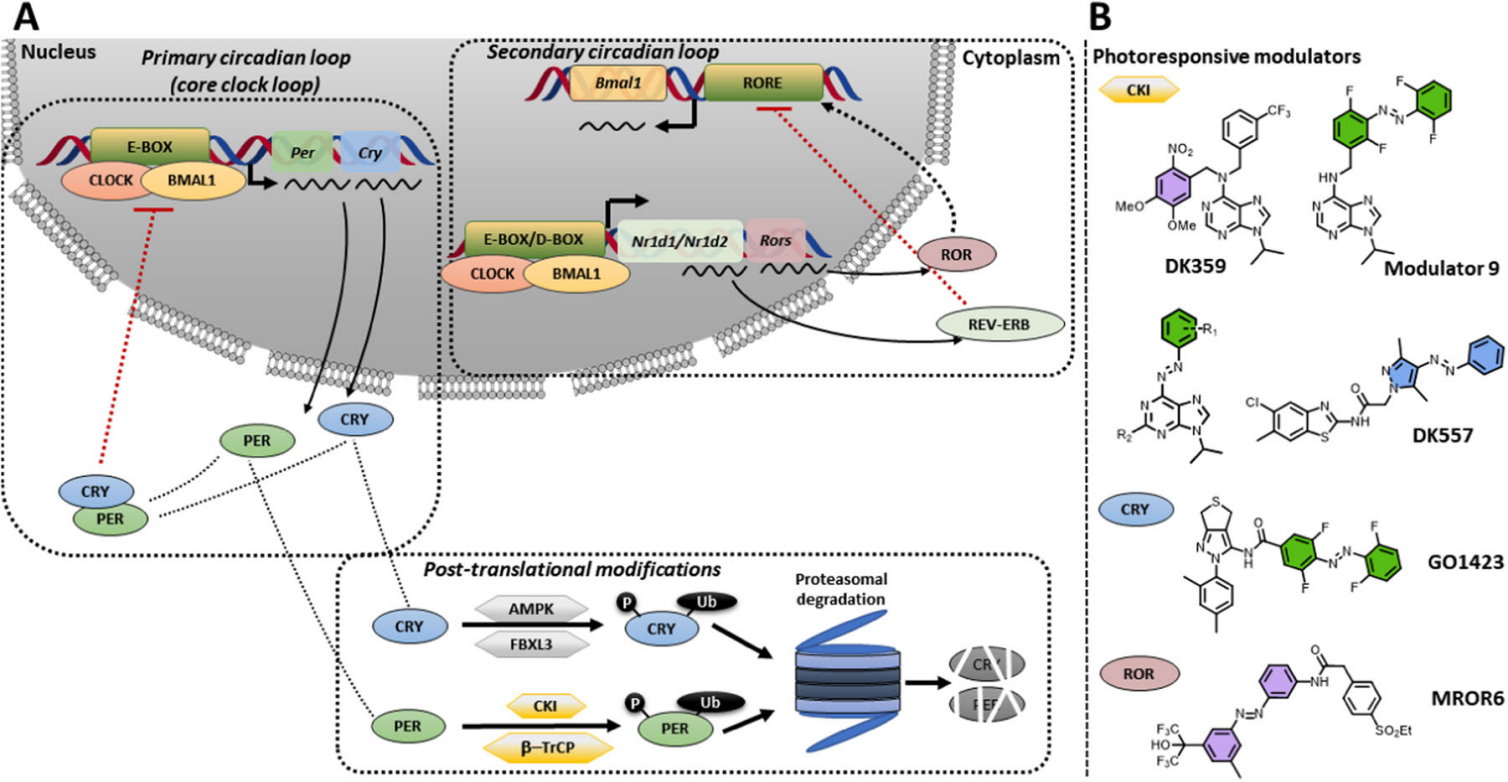
The endogenous regulation of circadian rhythms in mammals and an outline of small light‐responsive molecules that can be used for its reversible modulation. (A) Circadian regulation on the molecular level consisted of the primary and secondary circadian loop as well as posttranslational modification. Genes are denoted in italics, while promotors, proteins, and enzymes are in capital letters. (B) Photoresponsive modulators of the circadian rhythm, color‐coded based on the color of light they respond to for activation.

At the molecular level, the 24‐h rhythm in mammals is regulated through a multitude of transcription‐translation feedback loops (TTFL) of the clock genes. In this review, the focus lies on the loops that have been photo‐controlled [8, 34, 35]. Heterodimers of the brain and muscle Arnt‐like protein 1 (BMAL1) and circadian locomotor output cycles kaput (CLOCK) transcription factors make up the activator arm. In contrast, the repressor arm consists of cryptochrome (CRY1 and CRY2) and period (PER1, PER2, and PER3) proteins (core clock loop, Figure 3) [8]. After transcription and translation of *Bmal1* and *Clock* genes, BMAL1 and CLOCK proteins heterodimerize in the cytoplasm and translocate into the nucleus, where this complex binds to the regulatory elements Enhancer Box (E‐Box) and promotes the expression of *Per* and *Cry* genes. The resulting accumulation of PER and CRY proteins in the cytoplasm leads to their heterodimerization, followed by translocation into the nucleus, where they inhibit the BMAL1:CLOCK complex and, consequently, their expression. To create a feedback loop and restore gene expression, posttranslational modifications ensure the degradation of the inhibitory complex. At the posttranslational level, PER and CRY degradation is regulated through phosphorylation by casein kinases I (CKIα, CKIδ, and CKIε) and adenosine 3′,5′‐monophosphate (AMP) kinase (AMPK), followed by ubiquitination involving β‐Transducin Repeat Containing E3 Ubiquitin Protein Ligase (β‐TrCP) and F‐Box and Leucine‐Rich Repeat Protein 3 (FBXL3), respectively (posttranslational modification, Figure 3). Once the degradation of repressors progresses, the negative feedback loop is relieved, and BMAL1:CLOCK can start a new transcription cycle.

Next to the core clock loop, the secondary circadian loop reinforces the rhythmicity of circadian oscillations and makes them more robust (secondary circadian loop, Figure 3). This auxiliary loop consists of the ligand‐regulated transcription factors REV‐ERBα and REV‐ERBβ (encoded by *Nr1d1* and *Nr1d2*, respectively), as well as the retinoic acid orphan receptor (RORα, RORβ, and RORγ). The binding of the BMAL1:CLOCK complex to promoter regions containing E‐BOX and D‐BOX activates the production of REV‐ERBs and RORs. REV‐ERB and ROR compete for the same DNA response element called retinoic acid receptor‐related Orphan Receptor Element (RORE), which is responsible for the expression of *Bmal1*. While REV‐ERBs repress the expression of *Bmal1* upon binding to RORE, RORs are positive regulators, causing the BMAL1 protein level to increase.

All the aforementioned transcription factors and response elements affect the circadian clock genes and, simultaneously, the expression of numerous other genes whose promoters are E‐BOX, RORE, and D‐BOX. Therefore, by controlling the pace of circadian rhythm, the expression of specific genes can be tuned and directed to influence the prevention and development of particular diseases and disorders.

High‐throughput screenings have enabled the testing of small molecule libraries to identify potent circadian clock modulators [36, 37]. As shown in Figure 3, proteins involved in the core feedback loops and various posttranslational modification‐introducing kinases present potential druggable targets. Hence, here we distinguish two pharmacological strategies for circadian clock modulation: direct interaction with feedback loop proteins such as CRYs, PERs, RORs, and REV‐ERBs [38, 39, 40, 41, 42, 43], or targeting posttranslational modifications, predominantly through Casein Kinase I (CKIα, CKIδ, CKIε), but also Casein Kinase 2 (CK2) and Glycogen Synthase Kinase 3 (GSK‐3δ) (their contributions in TTFL were not described above given the scope of photopharmacologically targeted proteins) [37, 44, 45, 46, 47]. Small‐molecule modulators of circadian rhythm impact circadian oscillation parameters such as the period lengthening or shortening [37, 39, 44, 48, 49, 50, 51], amplitude increase or decrease [40, 52, 53, 54] and phase shift [52, 55]. While circadian amplitude and phase modulators exist, most small molecules influence the period, mainly by lengthening it. Modulating the period is beneficial, considering that changes in the circadian period cause sleep disorders and that cell proliferation in some cancer types can be stopped by clock inhibition [56, 57, 58, 59]. Despite many benefits that small molecules could offer to pharmacological chronotherapy, clinical investigations of these modulators are still scarce. Some significant obstacles are complex and intertwined circadian regulation networks that require further study by dissecting molecular pathways and, notably, uniform cellular regulation throughout almost the entire mammalian body. Thus, examining regulatory circadian pathways or fixing a locally disrupted clock using small molecules in view of the traditional pharmacological approach would be severely hampered by limited‐to‐non‐existent spatial and temporal selectivity. The lack of selectivity means that the potential drug would be systemically active throughout the body, and while it would fix the disrupted clock, the other healthy ones would be misaligned (pharmacological approach, Figure 2).

Inspired by one of the scientific breakthroughs—optogenetics [60, 61], the newly emerged field of chemical biology, photopharmacology [62, 63, 64, 65], has allowed circadian modulators to be rendered photoresponsive and achieve spatiotemporal regulation. Thus, in the future, it should be possible to use light to address regulatory pathways with a high degree of spatial and temporal control and treat only disrupted circadian clocks while minimizing the side effects on the healthy rhythms of other peripheral clocks (chrono‐photopharmacological approach, Figure 2). Given the merging fields of chronobiology and photopharmacology, this approach is referred to as chrono‐photopharmacology.

### 1.4 Summary

Healthy 24‐h circadian rhythms are essential in maintaining homeostasis in cells, organs, and the whole organism, and their disruption is linked to numerous disorders and diseases. However, a uniform biochemical regulation mechanism challenges the pharmacological, organ‐selective treatment with small‐molecule modulators. Therefore, unconventional approaches are required to obtain spatiotemporal control over the activity, opening the door for photopharmacology.

## 2 Chrono‐Photopharmacology

### 2.1 Photopharmacology: Irreversibly and Reversibly Light‐Activated Bioactive Molecules

**Figure 4:**
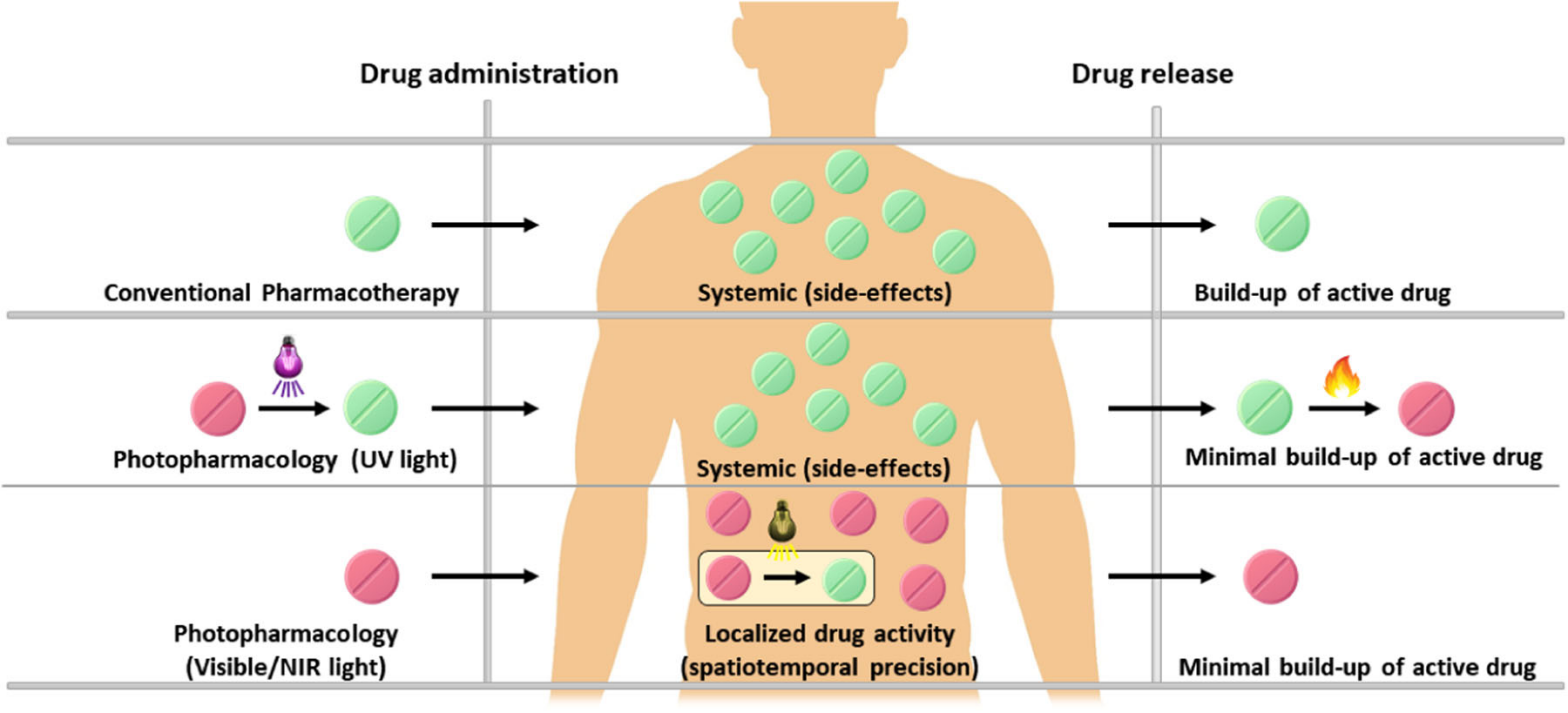
Principle of reversible photopharmacology. Adapted and modified with permission from Ref. [88] Copyright © 2017 American Chemical Society.

Photopharmacology has emerged as a field at the interface of organic chemistry, photochemistry, and medicinal chemistry [62, 64, 66]. The principle of photopharmacology is based on incorporating light‐responsive groups into existing drugs, allowing them to react to light at a structural level. Light is unmatched as an external stimulus due to its noninvasive nature and orthogonality to most biological processes [62] (Figure 4).

The two main strategies used to render a bioactive molecule photoresponsive are to modify it with a photo‐removable protecting group (PPG or “cage”) or a photoswitch moiety. The first approach results in an irreversibly light activatable drug, because light induces permanent structural changes, that is, PPG removal and release of the drug. In contrast, the second approach allows reversible modulation of biological activity with the light of different wavelengths, switching between two isomers (most commonly *cis* and *trans*‐configured C═C, C═N, and N═N double bonds). Both approaches, when applied to control circadian rhythm with light, constitute the field of chrono‐photopharmacology.

### 2.2 Chrono‐Photopharmacology Principles and Design Criteria

Many biological processes have been successfully addressed over the past decade using photopharmacology. Those include light‐induced regulation of bacterial communication and antibiotic resistance buildup, ion channels, G protein‐coupled receptors, kinases, lipid membranes, nucleic acids, and many others [62, 63, 64, 67, 68, 69, 70]. A common characteristic of these processes is that acquiring a desired response in biological assays is within (micro)seconds to a maximum of a few hours or 1 day. Conversely, circadian assays usually require five or more days to obtain an output signal that can reliably be analyzed [44, 52]. Along with this requirement, numerous other parameters must be considered and optimized at the molecular level to obtain dynamic control over circadian rhythm using photoresponsive moieties. While some of these challenges are common for photopharmacology in general, some are specific to chrono‐photopharmacology, and together, these are analyzed in the following paragraph.

**Figure 5:**
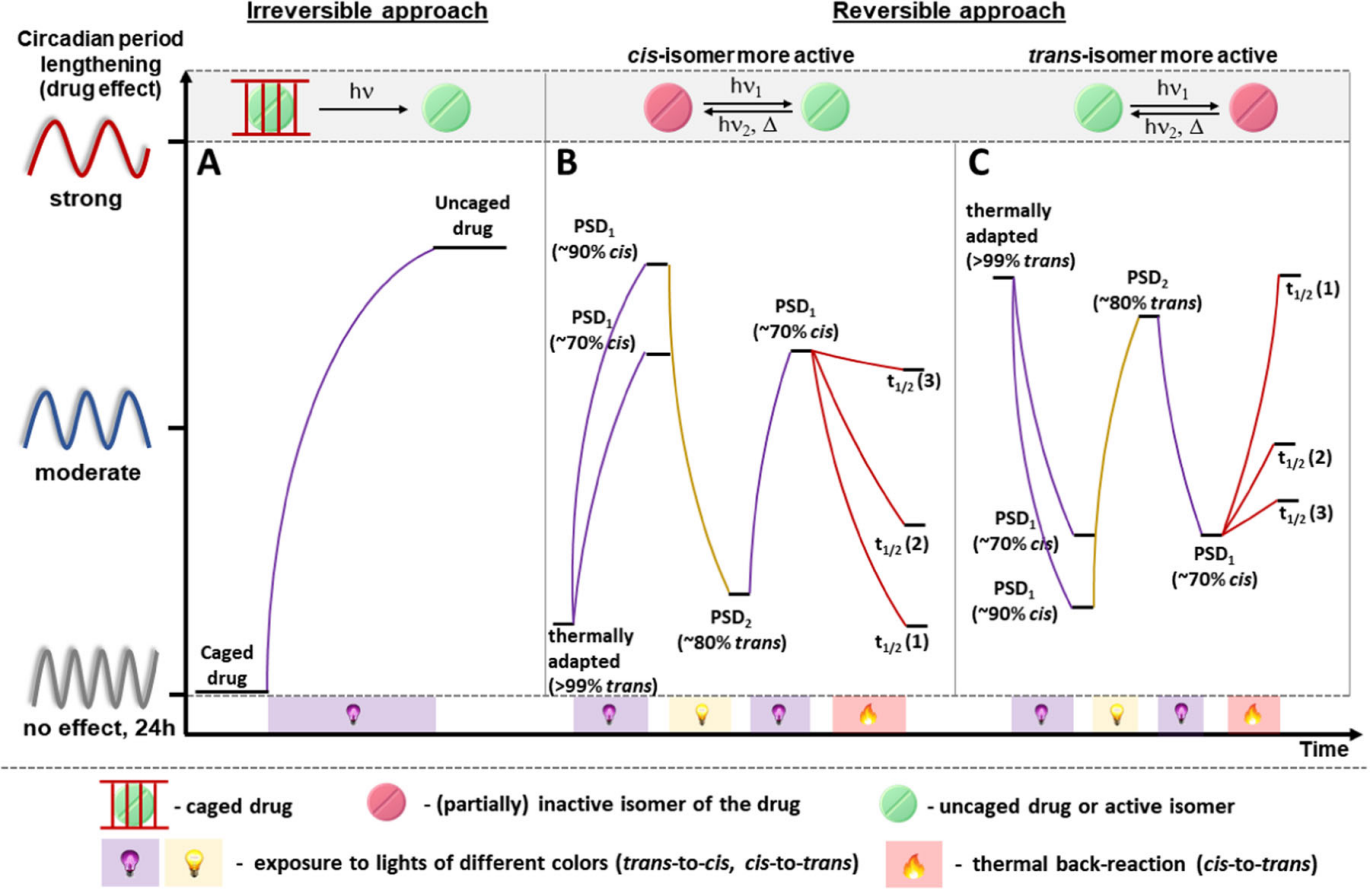
Scheme of (A) irreversible and (B, C) reversible approach to circadian rhythm modulation. The uncaging and *trans*‐to‐*cis* isomerization are indicated with a purple bulb, *cis*‐to‐*trans* isomerization using a yellow light bulb, and the thermal back‐isomerization in orange.

1. *Retaining potency, selectivity, and aqueous solubility upon incorporation of the photoswitch*. The highly optimized nature of drugs means that incorporating photoresponsive moieties will often result in a loss of potency. The reduction or complete absence of potency is the goal of the irreversible approach because the active parent molecule is released upon light irradiation of the caged inactive modulator (Figure 5A). However, in the case of photoswitchable modulators, potency is not easily restored or suppressed by exposure to light, and thus, the incorporation of the photoswitchable moiety requires elaborate and rational structural design [71].
  The additional structural motifs often reduce the solubility of a parent molecule in addition to potency. Reduced solubility makes it difficult to reach an effective pharmacological concentration, especially when a decrease in potency accompanies a decrease in solubility. This factor must be considered when applications in *ex vivo* or in vivo systems are envisioned.
  Drugs have also been optimized for selectivity to minimize off‐target effects, making it challenging to maintain target selectivity upon additional structural changes. This is particularly difficult for kinase targets due to the highly conserved catalytically active domain (the ATP‐binding pocket) across the kinome [72].
2. *Retaining tunable activity in biologically relevant media.* For example, a cell culture medium in which circadian oscillations are monitored requires an increased concentration of luciferin. This highly fluorescent organic compound challenges the utilization of UV light for in situ PPG removal and photoisomerization during assays. It inflicts the need for photochemical processes initiated by visible light (> 400 nm).
3. *Maximizing the difference in binding affinity between the ON and OFF states.* Due to the usually large group added to the drug structure, the affinity of the caged modulators is expected to be silenced. Upon light irradiation, the modulator is released, restoring its affinity. In the reversible approach, the difference in affinity between the two photoisomers should be as large as possible to achieve a distinct biological difference. Because the most commonly applied photoswitches, azobenzenes, rely on geometrical changes upon photoisomerization (the molecular constitution remains the same), achieving very high differences in binding affinities is challenging. As shown in Figure 5B,C, both isomers are typically active, but one has a more pronounced effect on period lengthening. Depending on the rational design [71] or serendipitous discovery, either isomer can be more active, and therefore, two cases are distinguished when observing the change in affinity starting with a more thermodynamically stable *trans*‐isomer: switching ON ‐ the less active *trans* isomer is converted to more active *cis* isomer (Figure 5B), or switching OFF the activity (the *cis* isomer is less active than *trans* isomer, Figure 5C). While maximizing the binding affinity difference between the isomers is important, it is worth mentioning that biochemical regulation relies on complex networks, leading to nonlinear responses upon target binding. This means that even smaller binding affinity differences towards a particular target could lead to a pronounced global alteration observed down the signaling cascade. Therefore, from the application point of view, it is often better to focus on optimizing other parameters discussed here than maximizing the binding difference to a single target.
4. *Achieving the highest possible photostationary state distribution (PSD)*. PSD stands out as a critical parameter in the design of photoswitchable drugs. It quantifies the practical efficiency of the photoswitching upon irradiation at a specific wavelength and is usually expressed as the ratio or percentage of one isomer to the other that can be achieved at the photodynamic equilibrium. For most photoswitches, photoisomerization fails to result in quantitative interconversion between isomers, leading to a particular PSD (PSD_1_, PSD_2_, Figure 5B,C). Since both isomers are typically biologically active, albeit with varying potency levels, achieving a high PSD becomes essential to minimize the background activity of the undesired isomer (e.g., consider the difference between ~70% and ~90% PSD_1_ in Figure 5B,C). Having a high PSD distribution is especially important when the thermodynamically stable *trans‐*isomer is more active, and the biological effect is suppressed by irradiation (Figure 5C). Due to commonly nonquantitative *trans*‐to‐*cis* photoisomerization, it is easier to observe the impact of the c*is*‐isomer if the *trans*‐isomer is (almost) inactive than to see its deactivation if the active *trans*‐isomer is present and active. Given the lengthy duration of circadian assays, starting with a high PSD and maintaining it as long as possible is crucial when measuring the effect of the metastable *cis*‐isomer. Failure to do so results in thermal relaxation during this extended period, which diminishes the effect of the *cis‐*isomer and hinders its quantification (for details, see point 5).
5. *Adjusting the thermal half‐life of the less thermodynamically stable isomer.* In most azobenzene switches, the *cis*‐isomer is thermodynamically meta‐stable and undergoes spontaneous thermal back‐isomerization to the *trans*‐isomer. In longer biological assays, even with the highest amount of *cis*‐isomer in the PSD, fast thermal back‐isomerization prevents the distinct effect of both isomers from being observed (*t*_1/2_ (1), Figure 5B,C). Generally, the faster the thermal isomerization, the lesser the impact of the *cis*‐isomer (the effect of t_1/2_ on the observed period lengthening is shown in Figure 5B,C). Conversely, the caged modulators are thermally stable.
6. *(Photo)Chemical stability in biological assays.* Avoiding oxidation or reduction of the photoresponsive moiety in a biological medium is crucial, especially if the effect is measured over extended periods, as in circadian assays. Isomerization is mediated by light, which acts as an external energy source. However, the energy input does not necessarily need to lead only to a desired photoisomerization reaction [73, 74]. For instance, some PPGs act as photosensitizers leading to the formation of singlet oxygen [75, 76], but this is commonly related to the niche of red‐shifted PPGs and this issue can often be overcome [77]. Fortunately, photoisomerization of the most commonly applied azobenzenes is on the time scale of picoseconds, preventing the formation of singlet oxygen [64].
7. *Using visible light for uncaging and photoisomerization.* Most biologically applied photoswitches and PPGs utilize UV light for uncaging and *trans*‐to‐*cis* isomerization. Although it is feasible to use UV‐light‐induced reactions for biological regulation, this is a limiting factor for application in ex vivo and in vivo studies. UV light is cytotoxic, has negligible tissue penetration, and is poorly biocompatible, whereas visible and infrared light are biorthogonal and have considerable tissue penetration. In addition, circadian bioluminescent assays usually rely on a luciferin‐rich medium that strongly absorbs UV light, preventing light‐induced reactions during the assay.
8. *Quantum yields.* The higher the quantum yields of the photoreactions, the faster these processes occur, leading to shorter irradiation times, fewer side reactions, and lower cytotoxicity.

**Figure 6:**
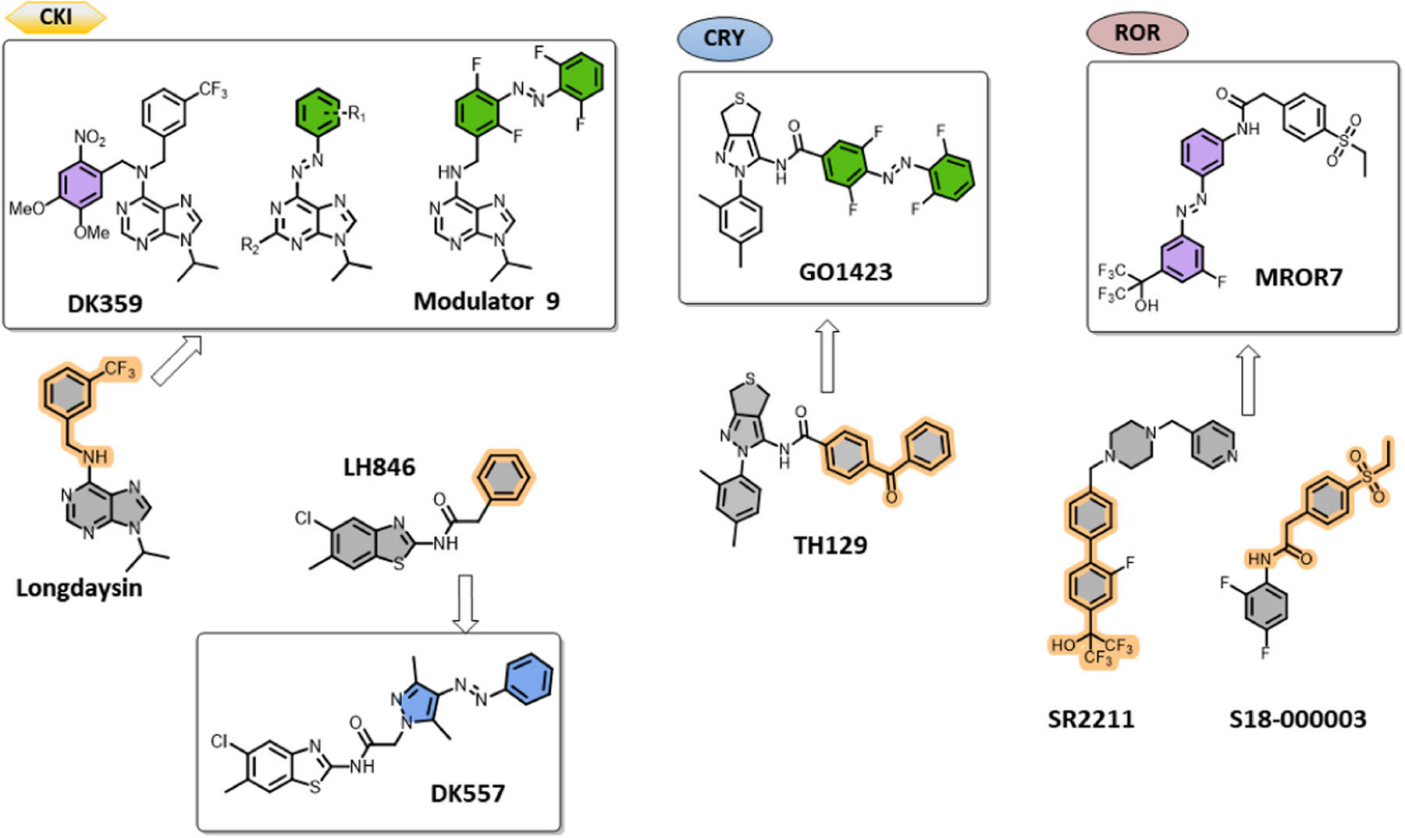
Photoresponsive modulators (violet, green, and blue) of the circadian rhythm and their parent structures (gray). Parts of the modulators used for creating the photoresponsive modulators are shown in orange, while the colors of the photoresponsive modulators refer to the wavelength of light they respond to.

This review summarizes and analyzes the development of photoresponsive molecules applied to all levels of biological complexity, from proteins to living organisms, and how their photophysical and photochemical parameters influence the modulation of circadian parameters (period and phase). To date, several photoresponsive systems have been reported to target posttranslational modifications and the core clock and secondary circadian loop. Light‐induced manipulation of posttranslational modifications was achieved by rendering Longdaysin and LH846 (CKIα and CKIẟ inhibitors, respectively) photoresponsive. The compound that selectively stabilizes CRY1 (TH129) and the inverse RORγ agonists SR2211 and S18‐000003 (among others) served as starting platforms for building photoswitchable analogues (Figure 6). At the outset of chrono‐photopharmacology, the scarcity of small molecules that interfere with the core clock loop directed the early research toward posttranslational modifications where CKI inhibitors and circadian period modulators were known [78, 79, 80, 81]. Later, reversible modulators of CRY1 and RORγ have emerged (Figure 6) [82, 83].

### 2.3 Light‐Controlled Protein Activity

**Figure 7:**
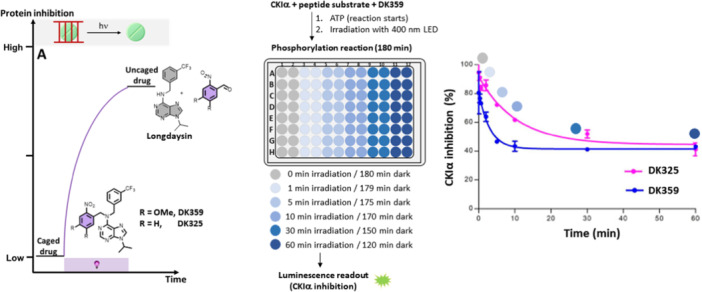
Irreversible approach to CKIα activity control: scheme of the approach and the experimental setup, as well as response curves of CKIα inhibition and irradiation time. Part of the figure was adapted with permission from Ref [80] Copyright © 2019 American Chemical Society.

The first reported control of circadian rhythms using chrono‐photopharmacology was based on the irreversible approach of caging Longdaysin (Figure 7) [80]. Thus, the introduction of an *ortho*‐nitro benzyl moiety (with and without methoxy substituents for a bathochromic shift ‐ **DK325** and **DK359**, respectively) at the 6‐NH position prevented the formation of two hydrogen bonds crucial for Longdaysin binding to the hinge region of CKIα and CKIẟ. Lack of hydrogen binding suppressed the inhibitory activity of Longdaysin in the dark. Upon increasing the irradiation time with violet light (400 nm LED, Figure 7), it was possible to dose Longdaysin through highly controlled release from its caged version (*photodosing*). Consequently, the inhibition of CKIα was also irradiation‐time dependent – the longer the irradiation, the lower CKIα activity was observed. Owing to the redshift of dimethoxy‐substituted PPG, **DK359** responded faster to violet light (Figure 7).

This irreversible approach was advantageously based on a straightforward synthesis offering modularity for introducing other wavelength‐superior PPGs. In addition, the solubility of the caged inhibitor was good because of the presence of a polar purine moiety. On the other hand, the main drawback of this approach is its irreversibility—once liberated, the inhibitor cannot be deactivated, reducing spatiotemporal resolution. Moreover, despite featuring clean photodeprotection (quantitative release of Longdaysin), all uncaging processes produce side products as leftovers of the PPGs, and these products could potentially be cytotoxic (albeit this was not observed in these experiments). Therefore, a reversible approach in which no chemical waste is produced, and activity can be controlled in both directions (activation and deactivation) would greatly benefit the spatiotemporal control of protein activity and circadian rhythm.

**Figure 8:**
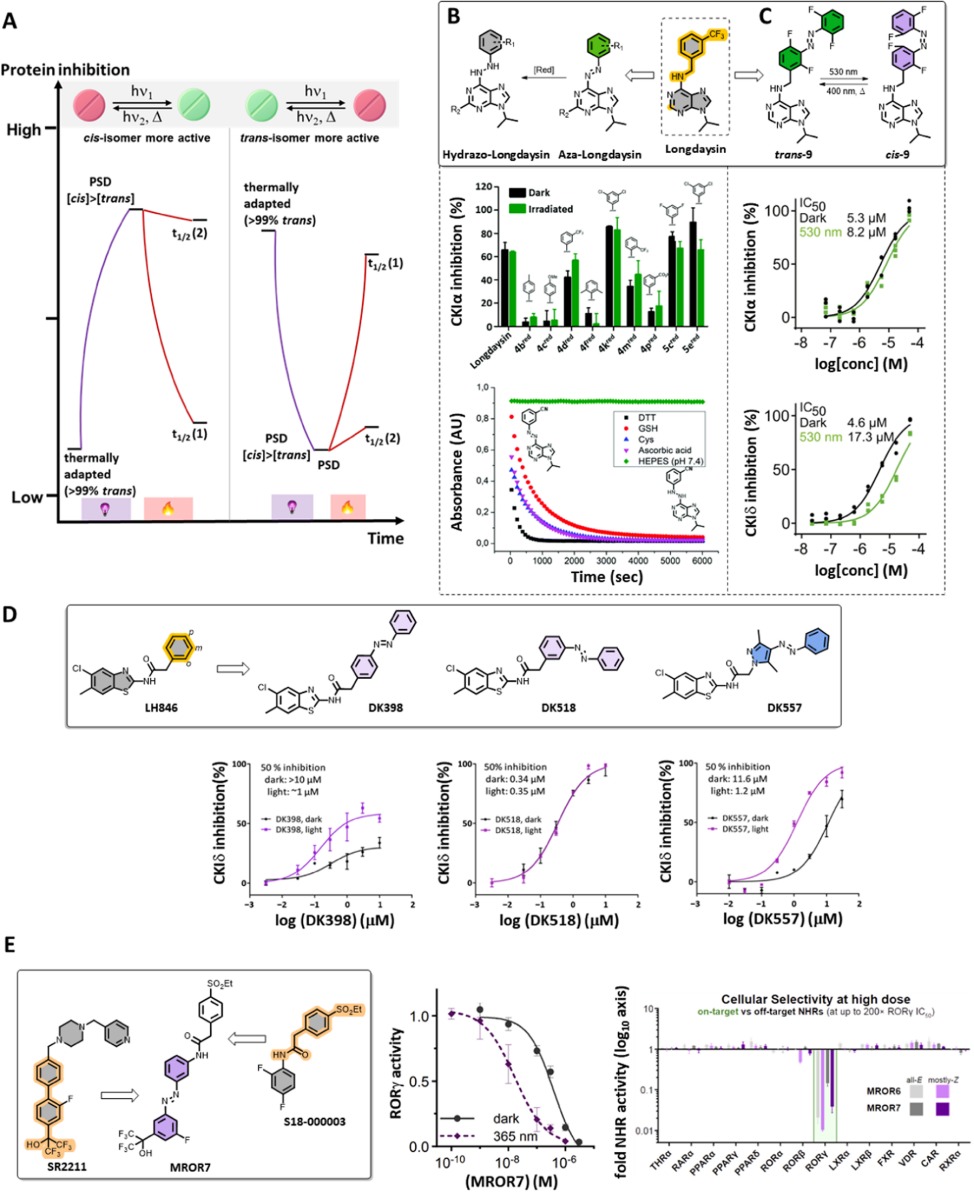
Control of CKI (CKIα and CKIẟ) and RORγ activity using photoswitches. (A) Schematic presentation of two possible enzyme activity modulation approaches using photoswitches – photoactivation and photodeactivation. (B, C) Two types of photoswitches based on Longdaysin inhibitor, their chemical properties, and protein inhibitions in the dark and upon light irradiation. (D) Light‐responsive CKIẟ inhibitors based on LH846. (E) Photoswitchable modulators of RORγ activity. Parts of the figure were adapted with permission from Ref [78, 79, 81, 83]. Copyright © 2021 The Royal Society of Chemistry, © 2021 Springer Nature, © 2022 MDPI, and © 2024 Wiley.

To enable reversible activation and deactivation of protein inhibition activity, the Feringa group designed photoswitchable small‐molecule inhibitors of CKI and CRY1 [78, 81, 82]. Recently, the Thorn‐Seshold group performed a reversible modulation of RORγ activity (Figure 8) [83].

The first generation of photoswitchable CKI inhibitors was envisioned to incorporate the azo moiety between the two aromatic cores of Longdaysin by substituting the benzylamine bridge (Figure 8B) [79]. A facile two‐step, one‐pot synthesis [84] allowed access to a library of green light‐switchable azo‐Longdaysins by different C2‐ (H or NH_2_, compound classes **4** and **5**) and C6‐ (R‐phenylazo) substitutions. Detailed photochemical analysis revealed moderate to good PSDs upon green light irradiation (*λ*_max_ = 530 nm, LED), short thermal half‐lives of the *cis*‐isomer, and lack of stability under reductive conditions in the presence of dithioerythritol (DTT, use in in vitro studies), glutathione (2–10 mM, GSH present in cells) and other reducing agents (Figure 8B). The short thermal half‐life (*t*_1/2_ (2), Figure 8A) and the fast formation of the corresponding hydrazo‐Longdaysins in the presence of DTT prevented measuring the *cis*‐isomer effect. While some of the compounds showed higher potency than Longdaysin (**4k**, **5e**) and a slight light‐dependent CKIα activity modulation (**4 f**, **5e**), this class of photoswitchable compounds clearly demonstrated the importance of high PSDs, long thermal half‐lives, and prolonged chemical stability for successful reversible control in long‐term biological assays.

Next, after an extensive optimization, the Feringa group developed compound **9** with all photophysical and photochemical requirements aligned with the circadian rhythm biological assays [78]. Compared to an electron‐poor azo bond in azo‐Longdaysins, adding the phenylazo moiety instead of the CF_3_ group of Longdaysin yielded a photoswitch resistant to reducing conditions. Especially the *ortho*‐tetrafluoro substitution of the azobenzene led to a long thermal half‐life (*t*_1/2_ > 50 h) and a good n→π* absorption band separation (~50 nm) of azobenzene isomers and thus enabled achieving high PSDs (86%) for both isomers under green (530 nm)/violet (400 nm) light irradiation [85, 86]. In *ortho*‐tetrafluoro azobenzenes, the *cis*‐isomer is stabilized, leading to a shift of the n‐orbital towards lower energy than the *trans*‐isomer. Better n→π* absorption band‐separation of the two isomers allows for using only visible light for photoisomerization (> 500 nm for *trans*‐to‐*cis* and > 400 nm for *cis*‐to‐*trans*, instead of commonly applied UV and visible light for the unsubstituted azobenzenes). Better band separation also leads to higher PSDs. Lastly, the stabilizing effect of the *ortho*‐substituents increases the energy barrier of Z→E thermal reversion, yielding azobenzenes with exceptionally long half‐lives. All these properties align with the required properties of photoswitches for applications in circadian assays.

For CKI activity photo‐modulation, thermally adapted (> 99% *trans*) and green‐light PSD (86% *cis*) were compared. The photoswitchable analogue retained potency compared to Longdaysin, with the *trans*‐isomer being 1.5 folds more active towards CKIα than the *cis*‐isomer (Figure 8C). Due to the slight difference in CKIα potency but strong photo‐modulation of the circadian period (see below), the same in vitro assay was performed on the CKIẟ isoform, revealing a 3.7‐fold difference between the isomers (Figure 8C). This established compound **9** as the first photoswitchable kinase inhibitor that, in its *trans*‐state, shows the same potency towards two isoforms (CKIα and CKIẟ), while upon photoisomerization, the *cis*‐isomer has higher potency for the CKIα isoform. Overall, it was possible to use only visible light to induce *trans*‐to‐*cis* (green) and *cis*‐to‐*trans* (violet) isomerization, revealing CKIẟ isoform as more critical in circadian period modulation.

For the development of a light‐responsive CKIẟ modulator, selective inhibitor LH846 was rendered photoswitchable (Figure 8D) [81]. First, the molecular architecture of the solvent‐exposed benzyl amide moiety was investigated to introduce the phenylazo group in a suitable way. While the *ortho*‐position (*o*, Figure 8D) was not synthetically accessible, *meta*‐ and *para*‐substitutions yielded two photoswitchable inhibitors, **DK398** and **DK518**. Both inhibitors retained their inhibitory activities and were stable under reductive conditions. The *meta*‐isomer displayed better solubility in aqueous media, but photo‐modulation of kinase activity was not observed. In contrast, **DK398** could not fully inhibit CKIẟ due to low solubility and decrease in potency upon azologization (vide supra), but it displayed a significant difference in inhibitory activity between “dark” and “light” conditions (all‐*trans* vs. *cis*‐enriched PSD). To increase solubility and retain photo‐modulation, the azobenzene moiety was replaced by arylazopyrazole (AAP) photoswitch with the same substitution directionality (**DK557**, Figure 8D). AAPs possess photochemical properties superior to those of azobenzenes—almost quantitative PSD (99% for **DK557** compared to 87% of the **DK398** *cis*‐isomer) and higher aqueous solubility, which was reflected in complete CKIẟ inhibition and a 10‐fold difference in potency between the isomers.

A recent publication from the Thorn‐Seshold group displays a light‐dependent RORγ inverse agonist prepared as a chimeric photoswitch built from two types of known RORγ ligands (Figure 8E) [83]. Initially, aiming that the *cis* isomer would better resemble the biphenyl moiety, azologization of a known inverse RORγ agonist—SR2211 (among other similar ones) was performed by incorporating the azo bond between the two phenyl rings. A series of five azobenzenes was prepared, with the *cis* isomer more active in all cases. The largest increase of activity upon UV‐light isomerization was 4.4‐fold. After further structural optimization by adding a polar substituent found in S18‐000003, the designed **MROR7** photoswitch exhibited outstanding potency (5 nM for *cis*‐enriched PSD) and selectivity against RORγ over related nuclear hormone receptors (NHRs). Moreover, the activity difference between the “dark” and irradiated samples was 20‐fold in the cellular reporter assay. Compared to other photoresponsive modulators of circadian rhythm, **MROR7** stands out as the one with the largest *trans*/*cis* activity change.

### 2.4 Modulation of the Circadian Period in Cells

**Figure 9:**
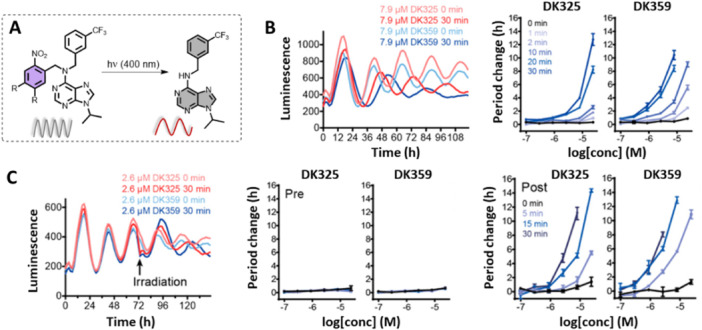
Irreversible control of the circadian period lengthening in cells. Modulating the circadian period in cells by precisely controlled irradiation (A) in the presence of caged Longdaysin modulators **DK325** and **DK359**. (B) Luminescence rhythms and the quantified period change of the U2OS cells treated with the caged modulators followed by dark conditions (0 min) or upon irradiation with violet light (30 min). (C) Circadian rhythms displaying the effect of violet light irradiation on Day 3 after the caged modulator was applied, and the corresponding period change during the first and second halves of the experiment. Parts of the figure were adapted with permission from Ref [80] Copyright © 2019 American Chemical Society.

To test the possibility of controlling the circadian period through photodosing, the Feringa and Hirota groups conducted experiments using human U2OS cells with the *Bmal1‐dLuc* reporter gene. Caged compounds (**DK325** and **DK359**) were applied to cells in a dose‐dependent manner, and the circadian period change in comparison to the control (ca. 24 h) was monitored during the increasing irradiation times (0–30 min, Figure 9A,B) [80]. Like in vitro assays, the caged compounds showed no period lengthening, while longer irradiation times caused extended period changes due to increased Longdaysin release and CKIα inhibition. Next, the possibility of modulating the circadian period on command was tested during the assay and not only from its beginning. The caged compounds were added at the moment zero and kept under dark conditions. During the course of 3 days, these compounds did not influence the period change, but when irradiated on Day 3, the luminescent signal revealed a significant period lengthening (Figure 9C). The latter experiment emphasized the chemical and biological stability and compatibility of the designed caged modulators and their entirely suppressed activity under dark conditions. This study illustrates control of the circadian period through photodosing, where fine‐tuning of the period was achieved (0.5 to more than 10 h) by irradiation with light.

Next to the irreversible approach, a few classes of photoresponsive modulators were also tested for reversible control over the circadian period in cells.

**Figure 10:**
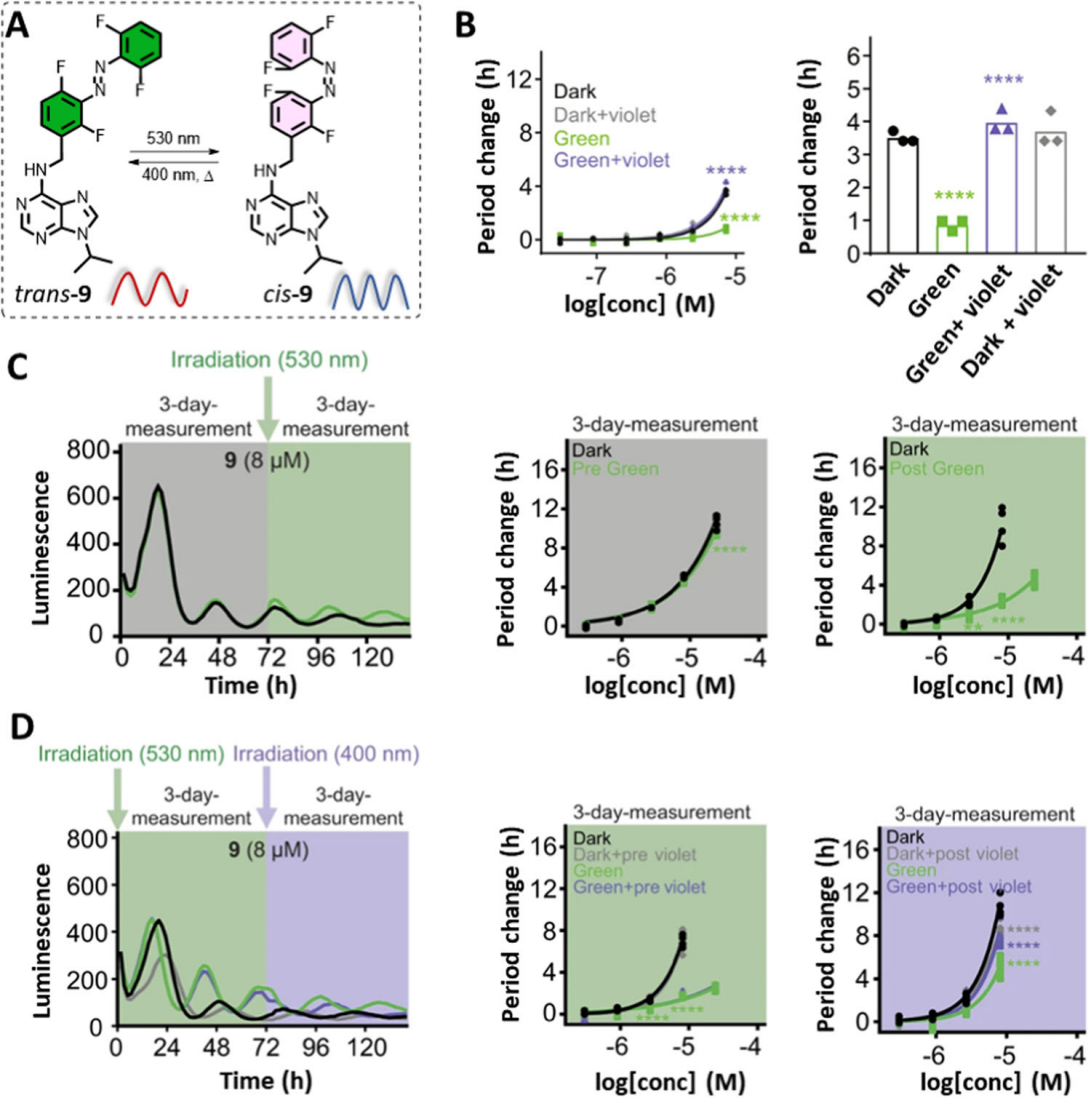
Reversible control of the circadian period lengthening in cells. (A) Photo‐ and thermal‐isomerization of compound **9**—*trans*, a potent modulator, and a weak *cis*. (B) Concentration‐ and light‐dependent period lengthening measured in U2OS cells. The modulator was applied to the cells and kept continuously in the dark (black), irradiated with green light to reach the corresponding PSD (green), irradiated with green followed by violet light (violet), and irradiated with violet light (gray). (C, D) The period change in a long‐term experiment where (C) the modulator was deactivated by green light on Day 3 or (D) deactivated with green light upon addition to the cells followed by reactivation with violet light on Day 3. Parts of the figure were adapted with permission from Ref [78] Copyright © 2021 Springer Nature.

While the attempt to modulate the circadian period using azo‐Longdaysin derivatives revealed some compounds with a stronger lengthening effect than Longdaysin, photo‐modulation was not possible due to their reduction to the corresponding light‐inactive Hydrazo‐Longdaysins (Figure 8B) [79]. On the other hand, by allowing the use of visible light without toxicity concerns and the limited tissue penetration depth characteristic of UV light, compound **9** enabled reversible circadian period modulation in cells containing a high concentration of luciferin (Figure 10) [78]. Interestingly, the *cis*‐**9** isomer exhibited weak period lengthening, while irradiation with green light yielded *trans*‐**9** with a more substantial period modulation (Figure 10A,B). Samples containing *trans*‐**9** (thermally adapted or *trans*‐**9** irradiated with violet or green followed by violet light) exhibited a period lengthening of ca. 4 h, while in situ photoisomerization to *cis*‐**9** with green light suppressed the period lengthening to ca. 1 h. This example also supports our statement (see subsection 2.2., point (3) that a large binding affinity difference toward a single target (in this case 3.7‐folds toward CKIδ) is not crucial for achieving a modular effect in cellular or other, more complex assays.

To demonstrate the power of the reversible modulator, which is visible‐light switchable and stable under prolonged reductive conditions, both isomers were tested with longer monitoring periods. *Trans*‐**9** and *cis*‐**9** (86% PSD) were added to the cells at the beginning of the 6‐day‐long experiment (Figure 10C,D). As expected, during the first 3 days of the experiment, the *trans*‐isomer strongly lengthened the period, while the *cis*‐isomer did it weakly. After the third day, the cells were briefly removed from the incubator and irradiated with green (*trans*‐to‐*cis* photoisomerization, Figure 10C) or violet (*cis*‐to‐*trans* photoisomerization, Figure 10D) light. Remarkably, the period‐lengthening effect of both isomers was altered, presenting the first photoswitch that could reversibly and in situ modulate the circadian rhythm during cellular assays.

**Figure 11:**
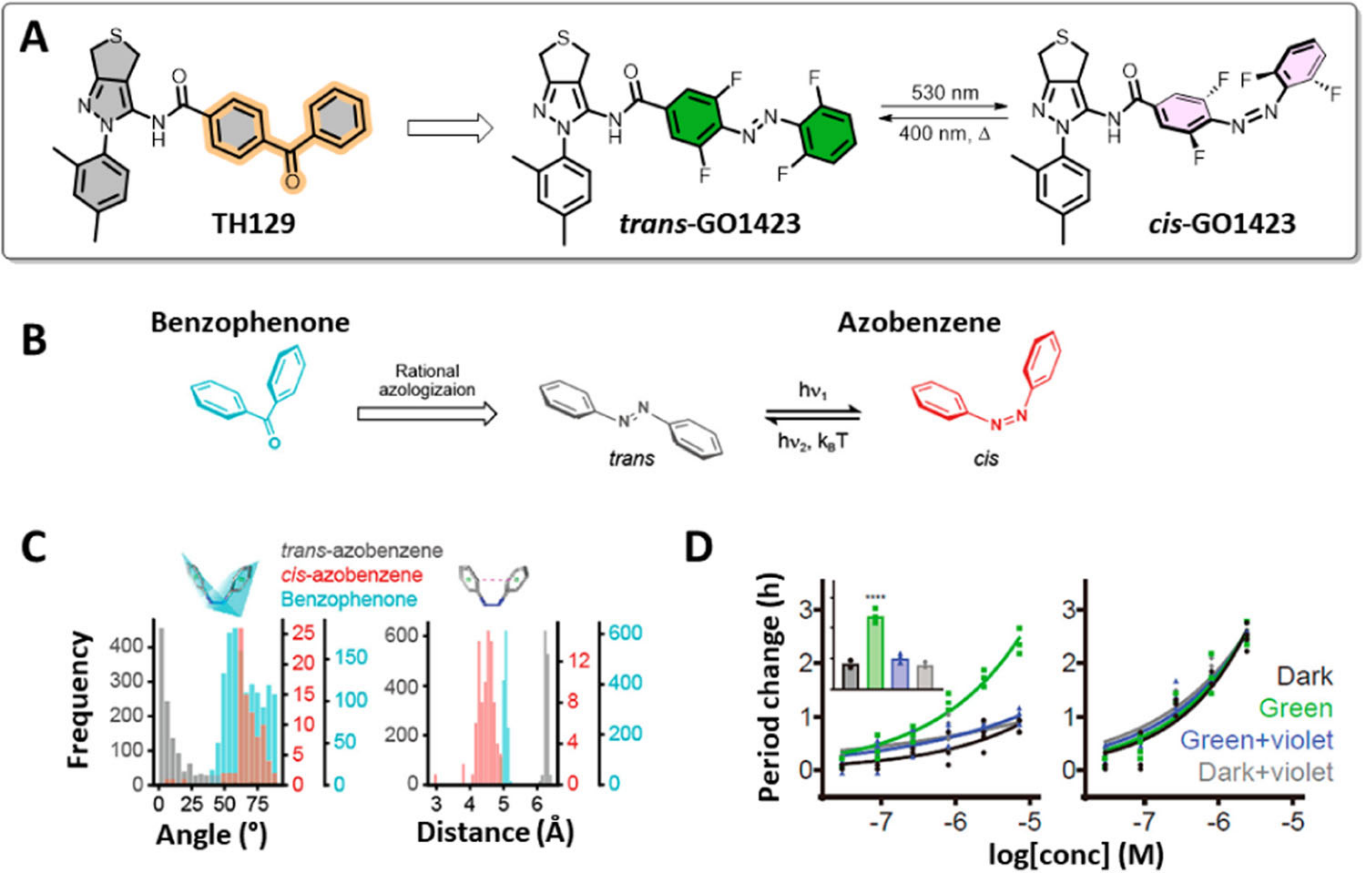
A reversible modulator of the circadian period based on the CRY1 selective binder, **TH129**. (A) Azologization of **TH129** and photoisomerization of **GO1423**; (B) A rationale behind azologization of benzophenones; (C) The analysis of data from Cambridge Structural Database (CSD) and comparison between distributions of the ring angles and distances of benzophenones, *cis*‐ and *trans*‐azobenzenes; (D) Period lengthening of *trans*‐**GO1423** (“dark,” “green + violet,” and “dark + violet”) and *cis*‐**GO1423** (“green”) as well as the period lengthening of **TH129** under the same irradiation conditions. Parts of the figure were adapted with permission from Ref. [82] Copyright © 2021 American Chemical Society.

The recent photoresponsive modulator successfully applied in cells to regulate the circadian period was **GO1423**, based on a selective CRY1 binder, **TH129** (Figure 11A) [82]. This is the first report of the rational adaptation of the benzophenone to a photoswitchable moiety (Figure 11B). It was hypothesized that benzophenone is structurally and electronically more similar to *cis*‐rather than to *trans*‐azobenzene and sought to support this idea by screening the available X‐ray structures for the structural features of benzophenones and azobenzenes. The comparison of the ring angles and distances corroborated the concept because the mentioned parameters almost overlapped between benzophenones and *cis*‐azobenzenes (Figure 11C). Hence, a turn‐ON modulator (more active in the cis‐form) was created by replacing the benzophenone moiety with azobenzene. The thermally adapted (all‐*trans*, “dark”) sample exhibited a period lengthening of less than 1 h. At the same time, upon irradiation with green light, *trans*‐**GO1423** was converted to the *cis*‐isomer with a period lengthening of ~ 3 h. While the circadian period was successfully modulated in cells, the limited solubility of **GO1423** prevented further investigation in tissues and living organisms.

The effect of **MROR7** on cellular RORγ‐regulated gene expression was tested in HEK293T cells harboring the mCherry reporter [83]. The fluorescent output signal of the cells containing the *trans* isomer was almost unaffected compared to the DMSO control, but the signal of the cells treated with the *cis* isomer strongly and dose‐dependently decreased. However, despite promising biological results, no further analysis of circadian rhythm influence has yet been performed.

### 2.5 Tissue Period Modulation

**Figure 12:**
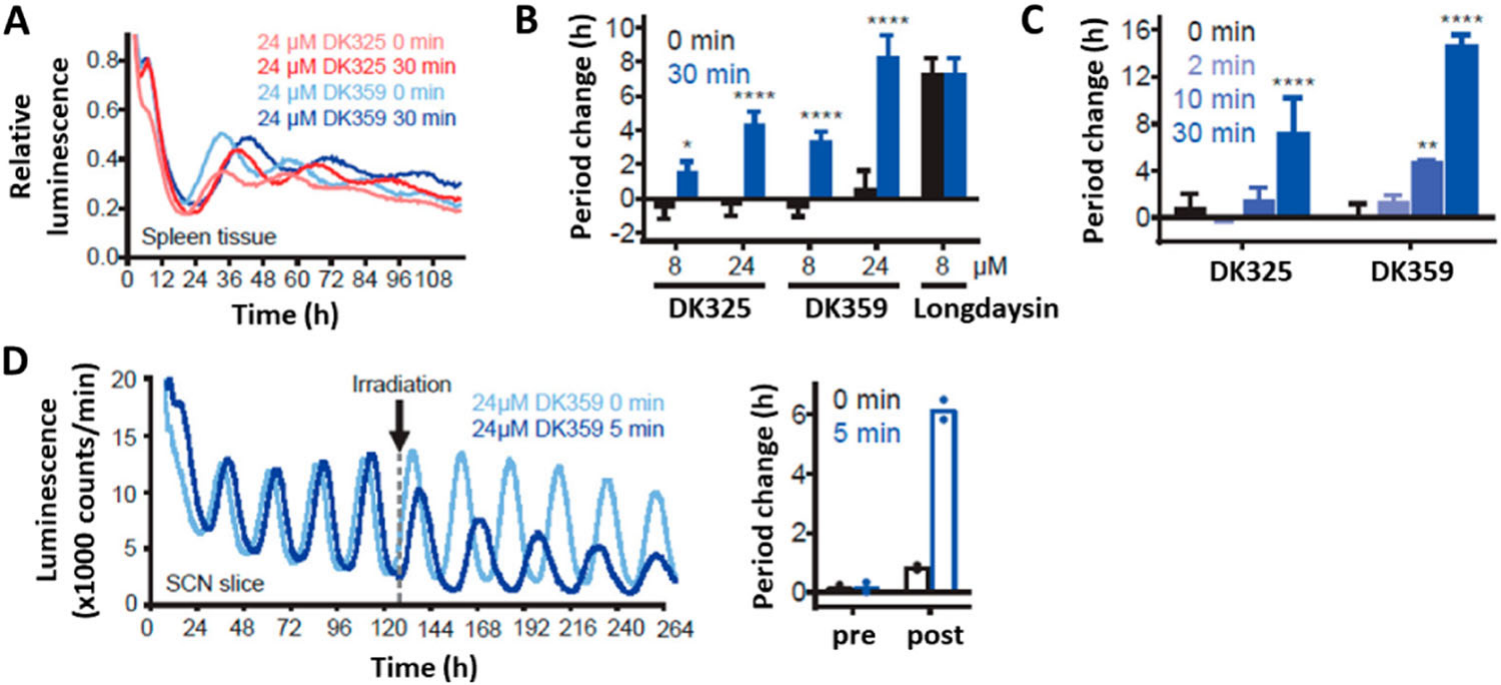
Photodosing of the circadian period in tissues. (A) Luminescence rhythms of the spleen explants in the presence of caged modulators (**DK325** and **DK359**) kept in the dark (0 min) or irradiated with violet light (30 min). (B) Quantified period lengthening of both modulators and Longdaysin in the dark and upon irradiation at two concentrations (8 and 24 µM). (C) The period change correlated with violet light exposure at a single concentration (24 µM, 0–30 min irradiation). (D) Luminescence rhythms and quantified period change in the SCN tissue explants. **DK359** was applied at the beginning of the assay and irradiated with violet light (5 min) on Day 5, whereas the other half was continuously kept in the dark. The figure was adapted with permission from Ref [80] Copyright © 2019 American Chemical Society.

Following the successful application of photodosing in cells, caged modulators were further evaluated in tissue application (Figure 12) [80]. Light‐induced period lengthening was initially demonstrated ex vivo using spleen explants from *Per2::Luc* knock‐in mice. Tissue explants were treated with the concentration range of **DK325** and **DK359**, and control samples were kept in the dark while others were irradiated with violet light (Figure 12B,C). Again, the results showed concentration‐ and irradiation‐dependent circadian period lengthening. A long‐term experiment was performed on SCN explants due to their robust and sustained oscillations over many days (Figure 12D). **DK359** was applied in the dark, and during 5 days, period lengthening was not observed. Subsequently, explants were irradiated with violet light, revealing a robust response of **DK359** to irradiation.

**Figure 13:**
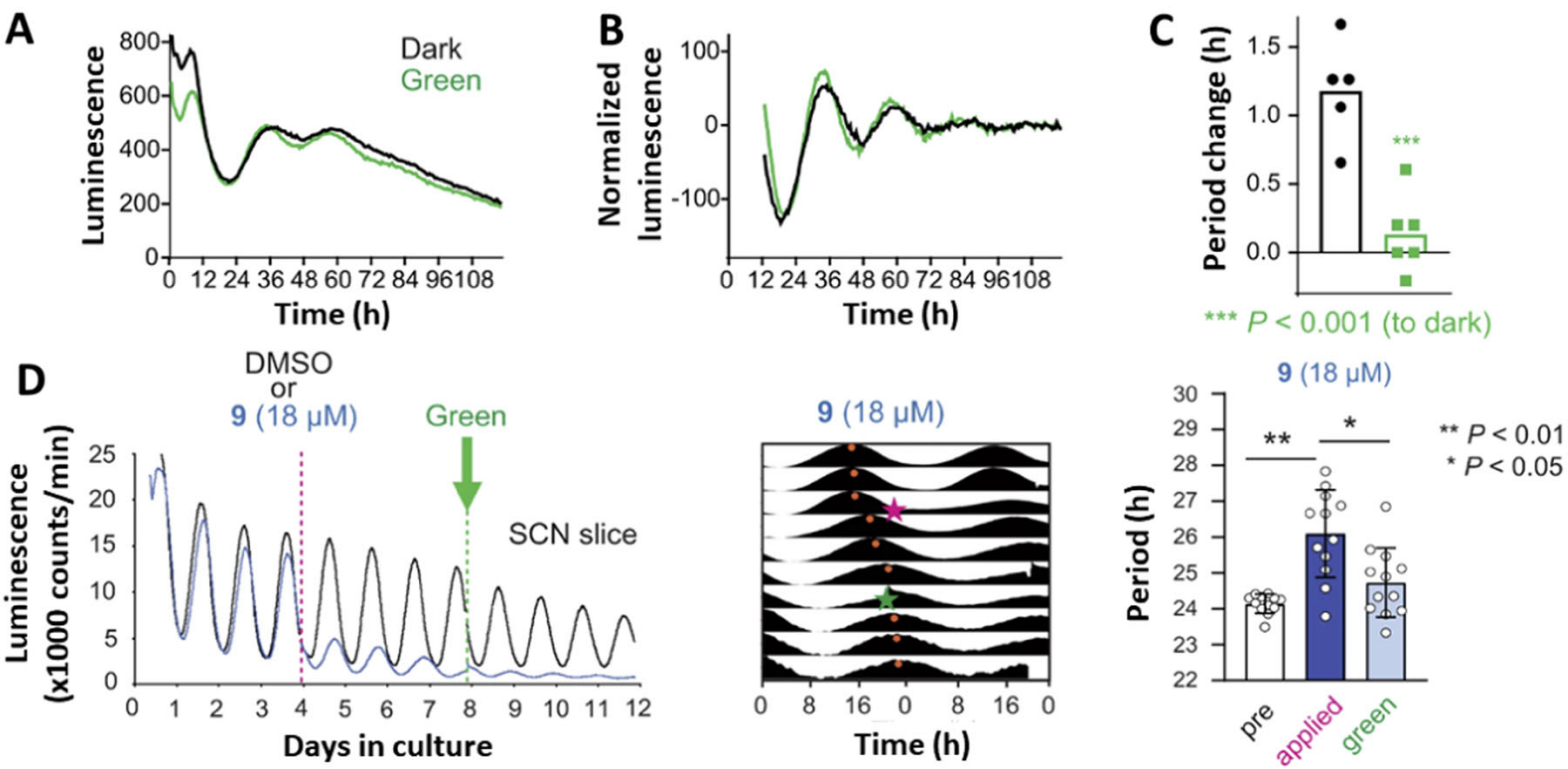
Reversible modulation of the circadian period in tissue. (A–C) The peripheral tissue (spleen) explants were treated with *trans*‐**9** and kept in the dark (black line) or irradiated with a green light at the beginning of the assay (green line). (D) SCN luminescence rhythms and the corresponding period changes. *Trans*‐**9** was added on Day 4, and the explants were irradiated with green light on Day 8 to suppress period lengthening. The figure was adapted with permission from Ref. [78] Copyright © 2021 Nature Springer.

Using the same experimental setup, reversible modulation of the circadian period was examined using modulator **9** at two different wavelengths of light (Figure 13) [78]. In both studies, a thermally adapted *trans*‐**9** modulator was applied to spleen explants. In one experiment, the explants were continuously kept in the dark, whereas in the other, they were irradiated with green light (86% PSD of the *cis*‐isomer). Consistent with the results in cells, the explants treated with *trans*‐**9** exhibited period lengthening (ca. 1 h), and upon photoisomerization to the *cis*‐isomer, this effect was suppressed (Figure 13A–C). Next, the reversible period control of the master SCN clock was investigated (Figure 13D). The potent *trans*‐**9** modulator was applied on Day 4, and a strong period lengthening was observed. Irradiation with a green light on Day 8 suppressed the period lengthening, bringing it back to almost 24‐h rhythms. These experiments illustrate the first reversible control of the circadian period during a long‐term assay using a (photo)chemically robust azobenzene‐based photoswitch **9**.

### 2.6 Temporal Control of Circadian Rhythms in Living Organisms

**Figure 14:**
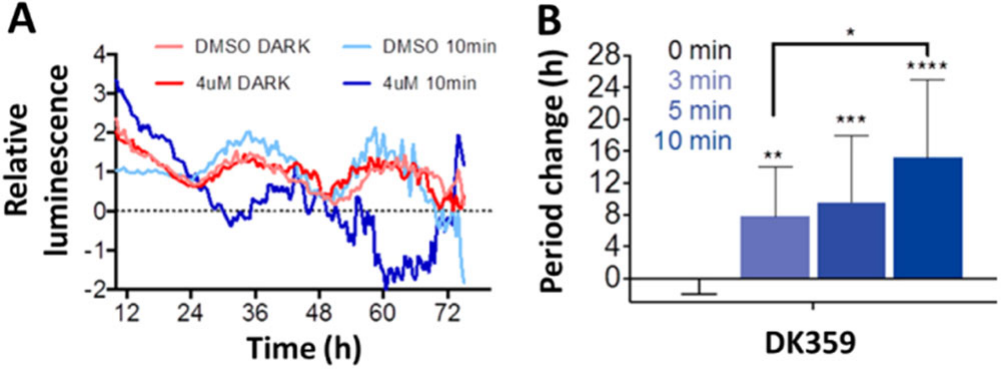
Modulation of circadian rhythm in zebrafish larvae. (A) Luminescence rhythms of larvae treated with DMSO or **DK359** followed by the dark or irradiated for 10 min with violet light. (B) Period change in larvae depending on the irradiation time. The figure was adapted with permission from Ref [80] Copyright © 2019 American Chemical Society.

To demonstrate the utility of the caged system, highly controlled temporal modulation of the circadian period was performed in living zebrafish larvae [80]. The samples were treated with **DK359** and irradiated with violet light (Figure 14). Similar to the cell and tissue experiments, the larvae kept in the dark did not indicate a period change, whereas the other rhythms were precisely modulated in an irradiation‐duration‐dependent manner (Figure 14B). These results, for the first time, indicate the ability to control circadian rhythms in living organisms using light as a clean external stimulus and a caged small molecule as a mediator.

### 2.7 Circadian Phase Modulation: The Power of a Reversible Chrono‐Photopharmacology

As previously mentioned, disruption of circadian rhythms can also be triggered by affecting the oscillation phase. For instance, jet lag is a typical example of circadian disruption caused by transmeridian travel that desynchronizes the phase of the internal rhythms from external day‐night cycles [87]. Thus, Feringa et al. hypothesized that using the reversible approach, a transient (temporal) period change could lead to an overall phase change, allowing for studying jet lag conditions or treating phase‐altered pathologies [78].

**Figure 15:**
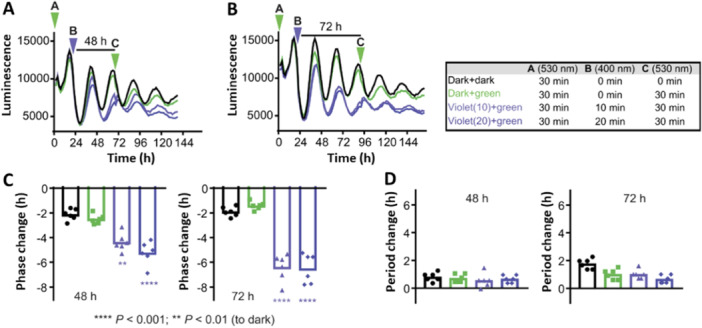
Circadian phase modulation with compound **9**. (A, B) Experimental design to transiently change the circadian period. (C, D) Quantifying the phase and period change. The figure were adapted with permission from Ref. [78]. Copyright © 2032 Springer Nature.

The experiment was designed to begin with a weak *cis*‐**9** modulator. Next, it was switched ON for 2‐3 days before being switched OFF again (Figure 15A,B). A detailed analysis of the period and phase revealed that the transient presence of *trans*‐**9** had a minimal effect on the period change while strongly influencing the phase (Figure 15C,D). This distinctive illustration of circadian phase modulation emphasizes the power of a reversible photopharmacological approach.

### 2.8 Summary

In this section, requirements for designing and applying photoresponsive modulators were summarized, with a particular emphasis on the desirable properties for creating light‐responsive modulators of circadian rhythm in mammals. Additionally, the current state‐of‐the‐art in chrono‐photopharmacology was discussed, as well as the corresponding parametrization of chemical, photophysical, and biological properties linked to successful applications. The work also gives an overview of current chrono‐photopharmacology probes within two approaches—irreversible and reversible, their advantages and disadvantages, and utilization across all levels of biological complexity for controlling protein activity and circadian rhythms in cells, tissue, and living organisms.

## 3 Conclusion and the Future Perspective

In the last two decades, photopharmacology has become a powerful method to spatially and temporally control biological processes with light. At the same time, chronobiology developed and provided new insights into homeostasis and health in mammals. Here, we aimed to present how these two fields synergize by summarizing recent discoveries in spatiotemporal control of biological rhythms using photoresponsive small molecules. The first caged and photoswitchable small molecule modulators of the circadian period and phase were developed, but many challenges remain. To be applied in mammals, photoresponsive modulators must be optimized for numerous photophysical, chemical, and biological properties. The most challenging chrono‐photopharmacological properties that need addressing are shifting the operating wavelength towards the red‐infrared range of the spectrum, increasing water solubility without solubilizing functional groups that impend cellular permeability, establishing photoresponsive modulators stable for days and highly potent, utilizing photoswitches with high (> 90%) or quantitative PSDs, and increasing the potency difference between the isomers. Currently, promising candidates for in vivo applications are compound **9** and **MROR7**. While **MROR7** has a low nanomolar potency that can be photoswitched by 10‐20‐fold, it remains unknown if these factors are sufficient to be translated to the ON‐OFF regulation of circadian rhythms. On the other hand, compound **9** has a lower potency with a 4‐fold difference between the isomers, but it is entirely visible‐light switchable, and the photo‐modulation effect in cells is pronounced. With these two examples, as well as using optimized modulators in the future, a lot can be learned about the complex biochemical oscillators that regulate circadian rhythms, the role of their disruption in disease and disorder development, and the challenges and prospects for future chrono‐photopharmacology. Lastly, circadian rhythms exist not only in mammals but also in plants, insects, fungi, and even some bacteria, which leads to the possibility of manipulating them using light‐responsive small molecules and learning about the interaction between these kingdoms of life, improving or inhibiting those of interest (e.g., addressing antibacterial resistance, plant growth, pesticide and drug efficacy and selectivity, etc.).

## Conflicts of Interest

The authors declare no conflicts of interest.

## Data Availability

The authors have nothing to report.
